# Understanding the diversity and biogeography of Colombian edible plants

**DOI:** 10.1038/s41598-022-11600-2

**Published:** 2022-05-12

**Authors:** B. Gori, T. Ulian, H. Y. Bernal, M. Diazgranados

**Affiliations:** 1 Royal Botanic Gardens, Kew, UK; 2 Pontificia Universidad Javeriana, Bogotá, Colombia

**Keywords:** Biogeography, Plant ecology, Plant domestication, Sustainability, Plant breeding

## Abstract

Despite being the second most biodiverse country in the world, hosting more than 7000 useful species, Colombia is characterized by widespread poverty and food insecurity. Following the growing attention in Neglected and Underutilized Species, the present study will combine spatial and taxonomic analysis to unveil their diversity and distribution, as well as to advocate their potential as key resources for tackling food security in the country. The cataloguing of Colombian edible plants resulted in 3805 species. Among these, the most species-rich genera included *Inga, Passiflora, Miconia, Solanum, Pouteria*, *Protium*, *Annona* and *Bactris*. Biogeographic analysis revealed major diversity hotspots in the Andean humid forests by number of records, species, families, and genera. The departments of Antioquia, Boyacá, Meta, and Cundinamarca ranked first both in terms of number of unique georeferenced records and species of edible plants. Significant information gaps about species distribution were detected in the departments of Cesar, Sucre, Atlántico, Vichada, and Guainía, corresponding to the Caribe and Llanos bioregions, indicating the urgent need for focusing investigation in these areas. Furthermore, a significant level of geographic specificity was found in edible plant species’ distributions between 13 different bioregions and 33 departments, hinting the adoption of tailorized prioritisation protocols for the conservation and revitalization of such resources at the local level.

## Introduction

Food represents the strongest form of interaction between humans and the environment. It lays at the foundation of human experience, shaping our relationship to other non-human living beings and embedding forms of intangible cultural legacy^1^. It is known that more than 7000 plants are edible^2,3^, meaning that “as a whole or their any part (roots, leaves or fruits) are acceptable for eating purpose by humans”^4^, p. 41). Many of them form part of the traditional gastronomic heritage of human populations and have the potential to support food security and develop sustainable agriculture around the world^3^. However, today almost the entire human caloric intake is made up of only ten species^5^. This incongruous trend was triggered by the green revolution^6^, which started to replace traditional landraces and wild species by a restricted assortment of modern commercial hybrids, favouring yield production^7^. The downsides of such direction were manifold. People started to diminish their interest in local edible plants while progressively decreasing their attention to the wellbeing of the ecosystems hosting them, which led to their degradation^8^. On the other hand, increasing pressure on a narrow portion of natural resources, together with unsustainable cultivation (e.g., substantial use of external agricultural inputs with impoverish the soil and damage pollinators’ populations) and harvesting practices (e.g., overharvesting of a given species in the wild), resulted in a rapid depletion of the natural populations of edible plants^9–11^. What is more, demographic growth and increased urbanisation resulted in severe land and forest cover changes: ongoing shifts towards urban centres caused progressive land abandonment^12^, and increasing demand for cereals, oils and meat led to the conversion of natural ecosystems to pastures and croplands^13^.

In response to this trend, international policy frameworks aimed at combining biodiversity protection and sustainable development gained attention over the last decades^14^. As reported by Borelli et al.^15^, efforts have increased to revitalize and promote the use of “orphan crops” and wild edible plants. Over the last few decades, more than fifty years after the green revolution, Neglected and Underutilised Species (NUS)—defined as “useful plant species which are marginalized, if not entirely ignored, by researchers, breeders and policy makers”^16^, p. 9)—have been proved to hold crucial importance for building sustainable livelihoods and mitigating environmental deterioration^15,17^, Ulian et al*.* 2020^3^). They also hold critical biocultural values, as they are linked to local agricultural systems and culinary traditions and practices, symbolizing the organic relationship between nature and culture. Growing evidence has demonstrated that the value peoples give to local plant resources can play a crucial role for their engagement in conservation and sustainable management^18,19^. “Conservation-through-use” approaches, aimed at encouraging nature conservation through the sustainable use of its resources, are increasingly being applied in conservation programs worldwide (Dulloo et al. 2017^20^, Oliveira Beltrame et al*.* 2018^21^).

Colombia is one of the world’s “megadiverse” countries^22,23^, hosting 10% of the global biodiversity^24^ and bringing together an unequalled number of distinct natural ecosystems and human cultures. However, despite its great biocultural richness, Colombia is nowadays characterized by widespread poverty, with more than 54% of its population suffering from food insecurity^25^. What is more, new land uses are now causing habitat destruction, driven by export oriented industrial agricultural policies and unsustainable market conditions^26,27^. While local communities had historically benefited extensively from local plant diversity (Rivas et al. 2010^28^), native plants consumption in the country has substantially decreased over time (López Diago & García 2021^29^). In fact, following the global trend, over the course of the past decades local edible plants have been rapidly replaced by high yielding commercial varieties^15^ and have become full-fledged NUS. Nevertheless, many underutilised species, although being rather unknown outside of the country, hold the potential to address environmental degradation, while creating sustainable livelihoods and boosting Colombian green growth^3,7^. Considering these circumstances, investigating and understanding Colombian Gastronomic Ethnobiology—the study of the complex interactions between people, food, and their environment^30^—acquires crucial importance for the formulation of targeted and effective conservation and sustainable development activities.

The first comprehensive documentation of Colombian useful plant diversity was conducted by Pérez-Arbeláez (1978), who catalogued 1771 species, including hundreds of edible species. Subsequently, Romero-Castañeda (1991)^31^ contributed extensively to the knowledge of Colombian edible fruits, cataloguing 167 species. In the following years, various taxonomic and ethnobotanical studies disclosed an even greater portion of the diversity of native edible plants, as well as their uses and socio-cultural values (Cf. Medina et al. 2019^32^;^33–39^). However, despite such valuable efforts, information on Colombian edible plants is still scarce. For instance, according to^29^, agricultural studies have been carried out on less than 20% of Colombian wild fruits. This constitutes a significant limit to the formulation of conservation-through-use strategies. Moreover, most ethnobotanical studies, being focused on a narrow portion of geographic locations, are not geographically representative of the entire Colombian territory. Indeed, the academic coverage of this topic leaves significant geographic gaps, an example of which is the almost total deficiency of research carried out in the Caribbean region^29^.

The Useful Plants and Fungi of Colombia (UPFC) project—started in 2019 by the Royal Botanic Gardens, Kew—aims at turning the potential of Colombian biodiversity into an economic resource for improving local livelihoods and food systems of impoverished communities. Locating itself within this fascinating and yet complex research framework, the present work aims to provide detailed information on the composition and distribution of the Colombian edible flora. We catalogued Colombian edible plant diversity and displayed a first prototype of its biogeographical distribution. Furthermore, we present useful information and knowledge for driving future efforts towards edible plants revitalization and conservation-through-use in Colombia.

## Materials and methods

Methods used for this study followed the approach employed by Diazgranados et al.^2^ for compiling the World Checklist of Useful Plant Species (WCUP) containing key taxonomic and ethnobotanical information on 40 292 species. Among all the plants classified as useful, 7039 were classified under the category of “Human Food”, following the Economic Botany Data Collection Standard^39^. The same taxonomic backbone of the World checklist of useful plant species was adopted here to reconcile taxonomically species coming from a combination of 10 new datasets and publications, both Colombian and international, in addition to the data coming from the Annotated Checklist of Useful Plants of Colombia^40,41^, New datasets were checked and cleaned in R 4.1.0^42^. Taxon names were reconciled to POWO^43^ and, when no such data were available, to Tropicos^44^ using “Plyr” and “Dplyr” packages^45,46^. Higher taxonomy information was obtained from the Global Biodiversity Information Facility^47^. Data on species edibility was retrieved from the cleaned datasets. Lastly, the number of Colombian edible NUS was obtained by checking the final dataset of Colombian edible species against FAO’s global census of agricultural crops^48^.

**Figure 1:**
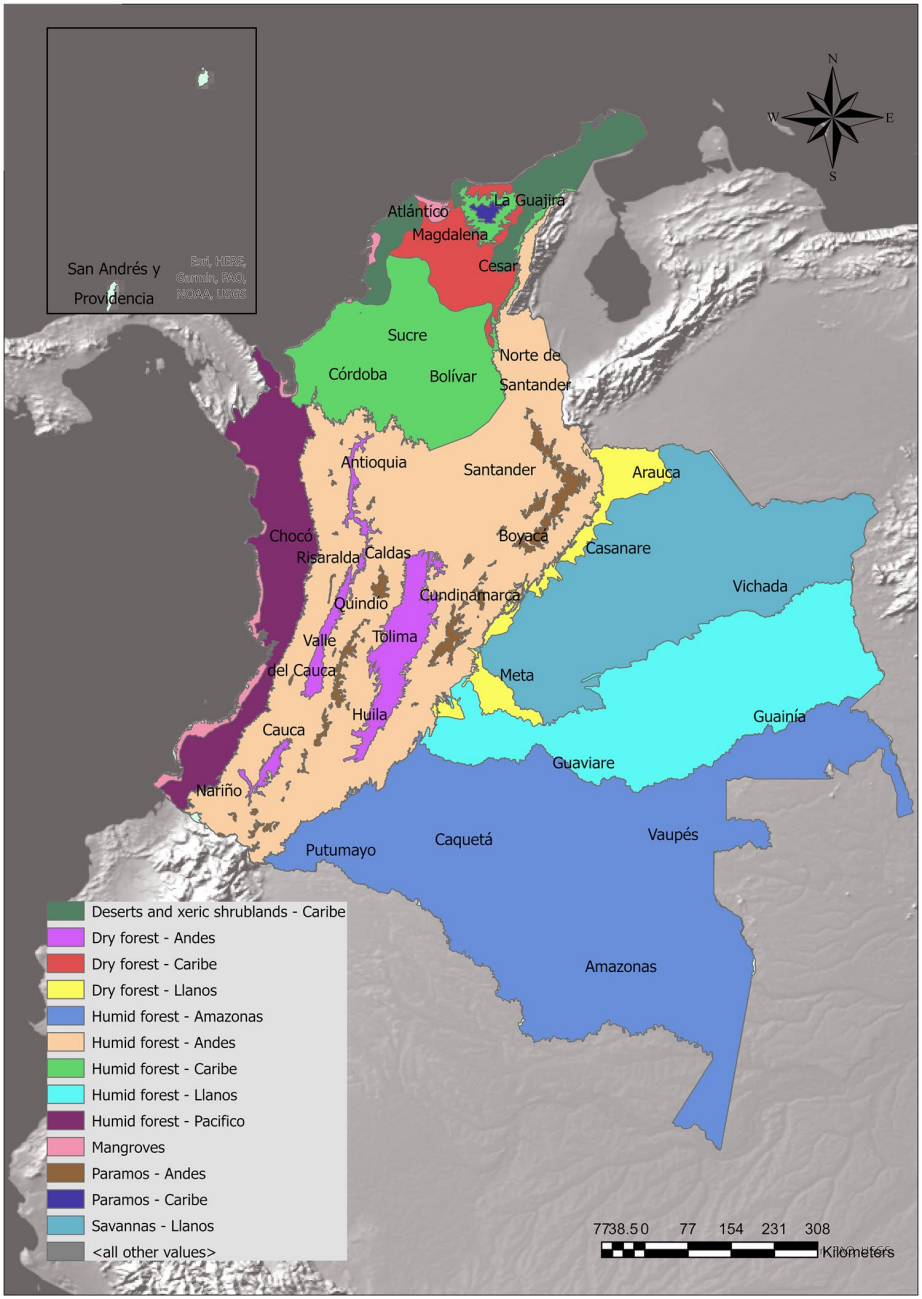
Overview of Colombian bioregions, generated in ArcGIS Pro 2.9.0. https://www.arcgis.com/index.html.

To contemplate both ecological and political factors affecting biodiversity distribution, we employed Colombian departments (32 departments and 1 capital district), bioregions (13) and cells (10 × 10 km^2^) as units for the present biogeographic analysis. We utilised a map of bioregions produced by Bystriakova et al.^49^, which combines the map of Terrestrial Ecoregions of the World (Olson et al. 2001^50^) with the five continental ecoregions in Colombia, resulting in 13 distinct units (Fig. 1). Unique georeferenced records of herbarium specimens for edible species were downloaded from GBIF (2021) through “RGbif” package^51^ and cleaned using “ShinyCCleaner” package^52^ in R 4.1.0^42^. Filters for removing occurrences recorded within urban centres, in the sea, within institutions (e.g., botanic gardens, ex-situ repositories), and in the centre of Colombia (i.e., centroid points) were applied. Moreover, latitude and longitude points with less than three decimals were removed, as well as occurrences with equal latitude and longitude and occurrences with either latitude or longitude equal to zero. Grid analysis to measure species richness was carried out using “rgdal” package^53^, “raster” (Hijmans and van Etten 2012^54^), and “sp*”*^55^. The complete resulting datasets are accessible in Figshare (https://figshare.com/s/cf5c19832ad4fd1695d7). ArcGIS pro 2.8.1 was employed to carry out biodiversity quantifications. SDMtoolbox^56^—a python-based toolbox for spatial analysis—was used to obtain biodiversity metrics such as species richness (i.e., sum of species per cell), weighted endemism (i.e., sum of the total number of cells each species in a grid cell is found, emphasizing areas rich in species with restricted distributional ranges), and corrected weighted endemism (i.e., weighted endemism divided by the total number of species in a cell, emphasizing areas rich in species with restricted ranges, but that are not necessarily species-rich), employing a geographic resolution of 0.1 degrees (~ 10 × 10 km^2^).

## Results

### Overview of Colombian edible plants

The current work resulted in the cataloguing of 3805 edible species (i.e., characterised by a history of consumption by human populations). Most edible species in Colombia are native (73.8%), and 457 are known to be naturalised (11.9%). Of these, 146 species (3.8%) are endemic (Bernal et al. 2020). Altogether, 662 species are currently cultivated (17.3%), and only 158 of them are native to the country. Out of 3805, the edible species reported by Colombian sources (i.e., reporting local food uses of edible species present in Colombia) are 2457. Thus, 1348 edible species are present in the country but do not have reported uses at the local level. A total of 117 species out of 3805 are mentioned in the FAO’s global census of agricultural crops. The remaining 3689, according to the definition of^16^, fall into the category of NUS.

Colombian edible plants are divided into 219 families and 1389 genera. Table 1 summarises the 20 most important families (i.e., containing the highest number of species). These include Fabaceae (119 genera/351 species), Asteraceae (86/136), Poaceae (72/140), Arecaceae (55/180) and Rubiaceae (52/137). However, if only native species are considered, families such as Melastomataceae (14/119), Malvaceae (35/91), Moraceae (18/74), Annonaceae (14/66) and Myrtaceae (11/61), obtain greater relevance. Important genera comprise *Inga* (84 spp.)*, Passiflora* (73)*, Miconia* (63) *Solanum* (61)*, Pouteria* (54), *Protium* (33), *Annona* (32) and *Bactris* (28). Genera such as *Ficus, Diospyros* and *Garcinia*, known to be among the most species-rich genera for edible plants at the global level^2^, are not significantly rich in edible species in Colombia. On the other hand, genera such as *Passiflora, Inga, Bactris* and *Pouteria* are characterised by a high number of edible species and may represent a new frontier for ethnobotanical and bromatological studies in the country.

**Table 1:** Top 20 families per number of genera recorded in Colombia, with count of native, cultivated, naturalised and endemic species.

Family	Count of Genera	Native species	Cultivated species	Naturalised species	Endemic species
Fabaceae	351	236	78	34	1
Arecaceae	180	132	34	0	12
Poaceae	140	53	32	50	0
Rubiaceae	137	115	9	2	4
Asteraceae	136	62	15	7	0
Malvaceae	129	91	27	2	8
Melastomataceae	123	119	1	0	3
Solanaceae	107	87	22	5	2
Myrtaceae	97	61	26	1	2
Sapotaceae	94	82	4	0	2
Moraceae	93	74	12	0	0
Passifloraceae	76	59	10	0	10
Annonaceae	71	66	1	0	4
Apocynaceae	65	50	6	3	0
Lamiaceae	64	23	20	13	0
Euphorbiaceae	62	47	11	3	0
Ericaceae	52	38	1	0	10
Urticaceae	52	43	6	1	0
Sapindaceae	49	42	2	0	2
Chrysobalanaceae	47	44	2	0	1

Colombian edible plants comprehend a great variety of growth forms, from trees to herbs, climbers, and epiphytes. Trees constitute the most dominant habit in terms of species richness, with more than 1500 species, followed by herbs, shrubs, and climbers. Growth habit highly reflects edible plants biogeographic distribution across various ecoregions (Fig. 3). Some of the most important tree genera include *Inga* (64 spp.)*, Pouteria* (49)*, Miconia* (39)*, Protium* (32)*, Annona* (27)*, Ficus* (21)*, Casearia* (21) and *Matisia* (20)*.* On the other hand, important herbs comprehend *Solanum* (30)*, Cyperus* (16)*, Miconia* (14)*, Oxalis* (11) and *Eragrostis* (11). Predominant genera for shrub species include *Miconia* (54)*, Solanum* (33)*, Bactris* (25)*, Casearia* (20)*, Piper* (15) and *Senna* (15)*.* Finally, most important genera for edible climbers comprise *Passiflora* (64)*, Ipomoea* (15)*, Dioscorea* (15)*, Paullinia* (14) and *Solanum* (12).

### Edible species distribution & diversity hotspots

The cleaned dataset of georeferenced records employed for the current analysis contains 221 838 georeferenced records for 3132 species, equal to the 82.3% of the total number of edible species present in Colombia. In fact, 673 species (17.6%) are not associated with any georeferenced record. Furthermore, only ten species make up the 24.4% of the total number of occurrences (e.g., *Acacia decurrens* (J.C.Wendl.) Willd.*,* 10 917 occurrences; *Eucalyptus globulus* St.-Lag*.,* 9557; *Pinus radiata* D.Don*,* 9391*; Cedrela odorata* L.*,* 4561*; Guazuma ulmifolia* Lam.*,* 4397*)*. Of them, the top three species are not native to Colombia. Overall, 2272 species (59.7%) are associated with 20 or less georeferenced records, and 1806 (47.4%) with 10 or less.

**Figure 2:**
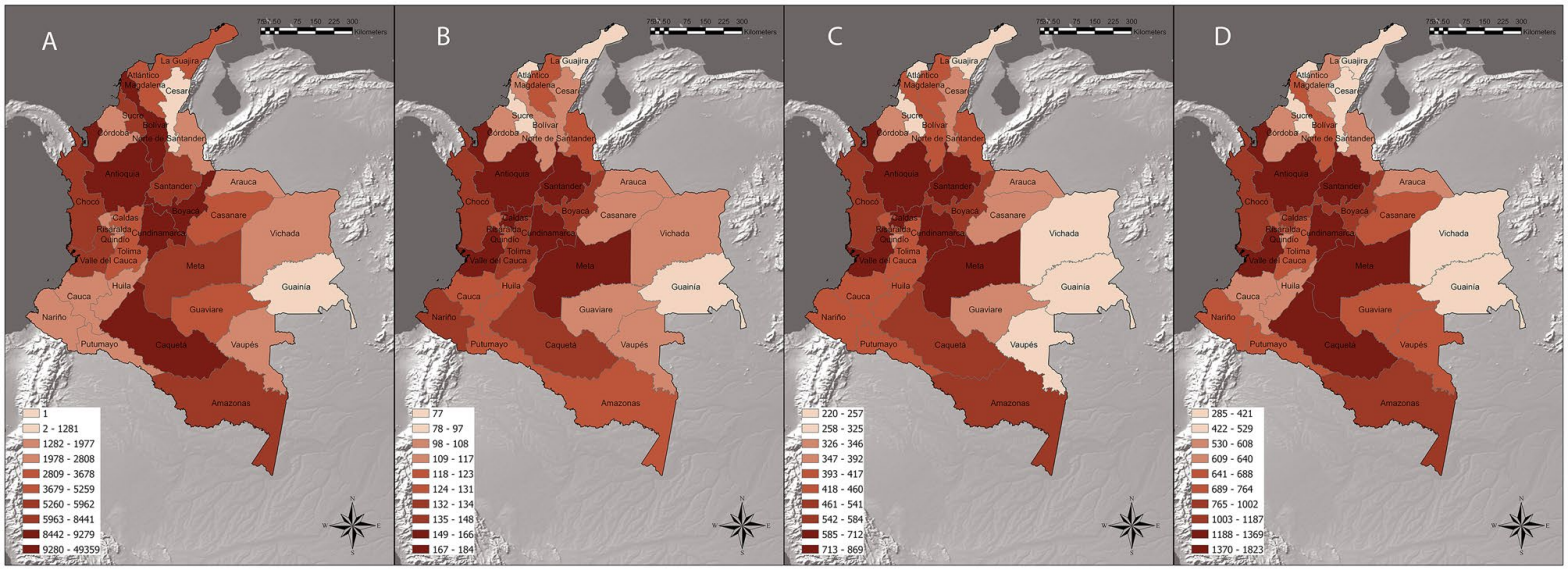
Distribution of Colombian edible plant species: (**A**) Number of unique occurrences per department; (**B**) Families richness per department; (**C**) Genera richness per department; (**D**) Species richness per department. Generated in ArcGIS Pro 2.9.0. https://www.arcgis.com/index.html.

The distribution of Colombian edible plants per department is showcased in Fig. 2. Results highlighted the department of Antioquia as the most diverse in terms of species (Fig. 2D), genera (Fig. 2C) and families (Fig. 2B), counting 43 696 individual georeferenced records (Table 2). Following it, the departments of Cundinamarca, Boyacá and Meta showed similar trends, totalling 49 359, 9192 and 8330 occurrences respectively (Table 2). On the other hand, departments such as Cesar, Arauca, Vichada and Guainía ranked last both in terms of species richness and number of occurrences, with no more than 1800 georeferenced records individually. Due to the complete absence of georeferenced records of edible plants, the department of San Andrés y Providencia was not included in the resulting figures.

**Table 2:** Number of unique georeferenced records of edible species, species, genera and families recorded in each Colombian department.

Department	Occurrences	Families	Genera	Species
Amazonas	7760	129	513	1187
Antioquia	43,696	184	869	1823
Arauca	1667	117	383	626
Atlántico	4539	77	220	285
Bolívar	9279	114	435	672
Boyacá	9192	148	566	957
Caldas	5259	162	682	1165
Caquetá	9088	141	584	1289
Casanare	3097	113	381	685
Cauca	1677	131	400	640
Cesar	1281	103	346	505
Chocó	5962	140	541	1002
Córdoba	2281	108	368	591
Cundinamarca	49,359	166	712	1275
Guainía	1064	94	257	416
Guaviare	3331	111	380	727
Huila	1976	123	404	625
La Guajira	4874	97	305	421
Magdalena	3678	129	417	626
Meta	8330	171	783	1624
Nariño	2733	134	431	764
Norte de Santander	1977	122	411	616
Putumayo	1802	123	409	746
Quindío	2808	122	392	608
Risaralda	2458	128	432	688
San Andrés y Providencia	1	0	0	0
Santander	8018	174	756	1369
Sucre	8441	95	309	446
Tolima	3274	134	460	751
Valle del Cauca	7746	160	669	1263
Vaupés	2666	103	325	681
Vichada	1711	101	312	529

**Figure 3:**
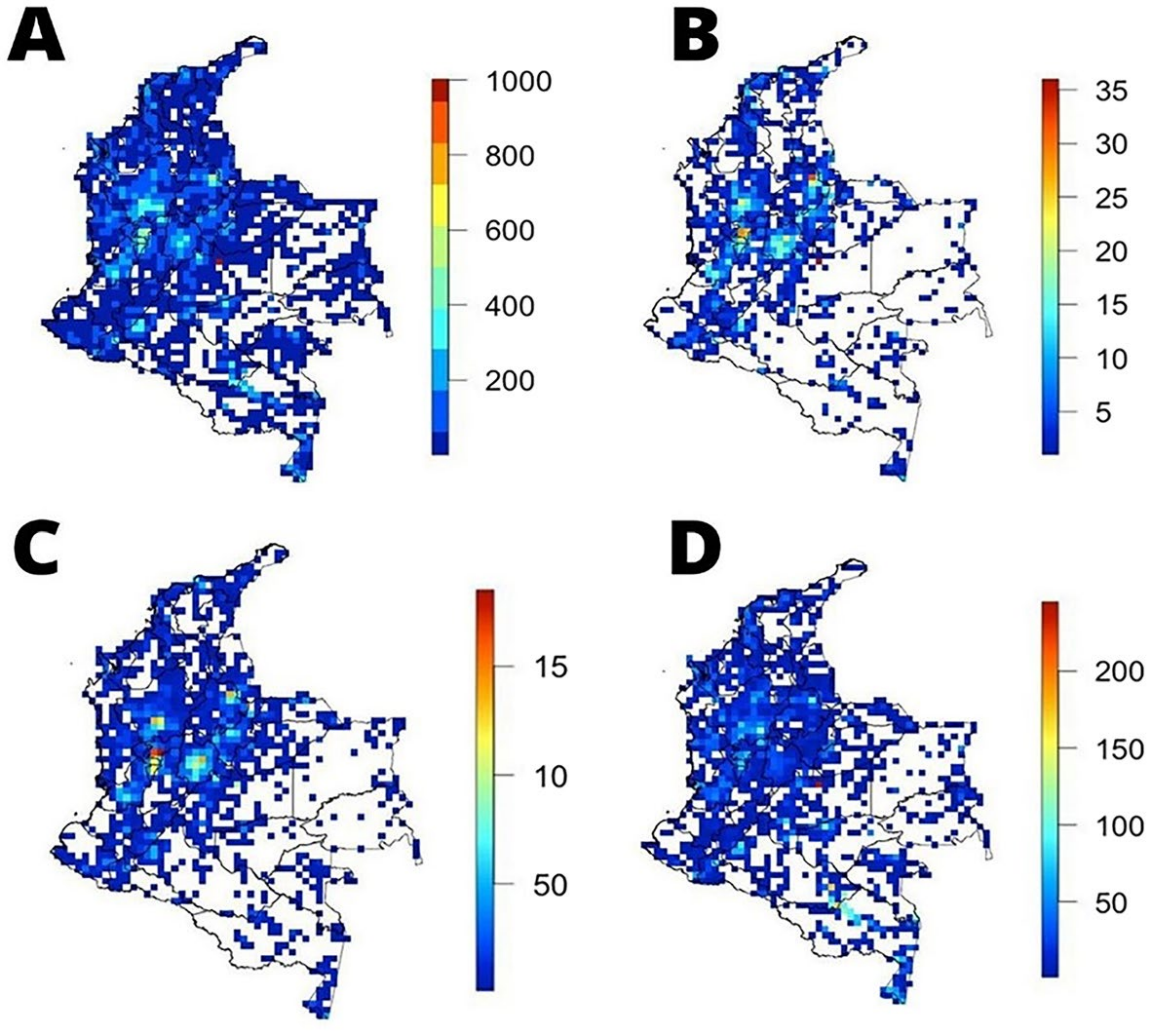
Distribution of Colombian edible plants by growing habit: (**A**) tree species, (**B**) shrub species, (**C**) herb species and (**D**) climbing species. The four distributions show relatively congruous trends, with high species concentrations across the Andean region. Generated in R 4.1.0. https://www.R-project.org/.

Across the country, 172 species (4.5%) are found in more than twenty departments, only fifteen species (0.3%) are found in more than thirty departments, and only one species—*Eleusine indica*, an introduced grass from the Tropical and Subtropical Old World*—*is found in thirty-three out of thirty-three departments. Only four of the species present in more than thirty departments (i.e., *Gynerium sagittatum* (Aubl.) P.Beauv., *Lasiacis procerrima* (Hack.) Hitchc. ex Chase, *Paspalum conjugatum* P.J.Bergius*, Setaria parviflora* (Poir.) Kerguélen) are native to Colombia, all belonging to the grass family (Poaceae), and seven species are currently cultivated (i.e., *Ananas comosus* (L.) Merr., *Bixa Orellana* L., *Cocos nucifera* L., *Mangifera indica* L., *Musa acuminata* Colla, *Saccharum officinarum* L., *Zea mays* L.). On the other hand, more than half of all Colombian edible species (2251; 59%) are specific to up to five departments, and 795 species (20.8%) have only been recorded in one department (Fig. 3).

**Figure 4:**
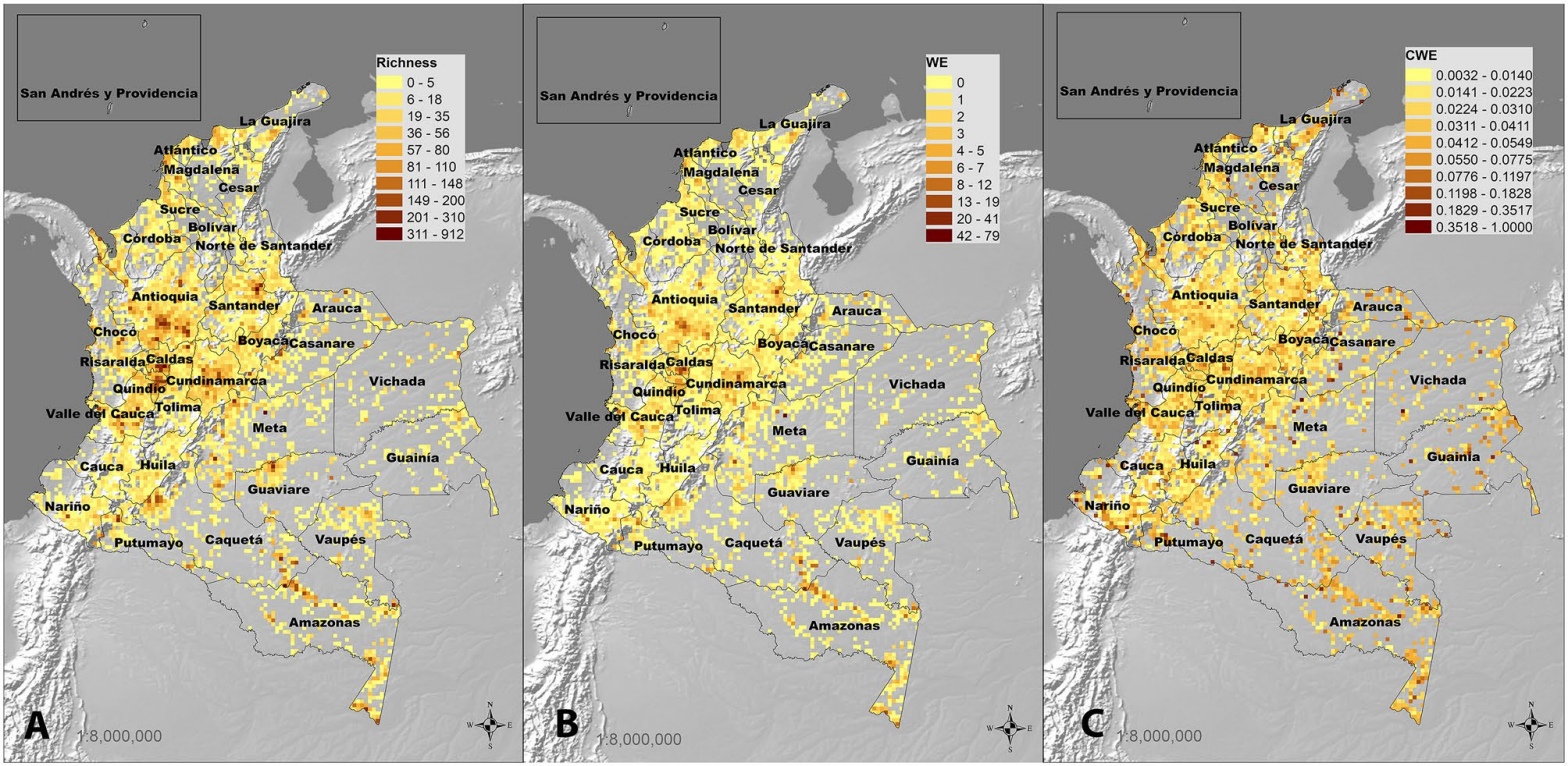
Diversity patterns of Colombian edible plants. (**A**) Species richness (SR) map; (**B**) Weighted Endemism (WE) map. (**C**) Corrected Weighted Endemism (CWE). Generated in ArcGIS Pro 2.9.0. https://www.arcgis.com/index.html.

Grid analysis revealed several hotspots for edible species diversity across the country (Fig. 4). Both the Species Richness (SR) and Weighted Endemism (WE) analysis emphasised the northern and north-western Andean region as crucial repositories of edible species (Fig. 4A, B). In particular, the highest SR was recorded between the areas of Antioquia, Boyacá, Cundinamarca and Caldas, which extend across three distinct bioregions: the Andean dry forest, humid forest, and páramo. In contrast, the Corrected Weighted Endemism (CWE), emphasising areas that are characterised by a high proportion of species with restricted distributional ranges, displayed a more scattered distribution of numerous restricted hotspots (represented by dark brown cells in Fig. 4C). A considerable density of narrow-distribution edible plants was recorded in the Amazonian bioregions, in the departments of Amazonas, Caquetá and Vaupés, as well as in the departments of Guainía, Nariño and La Guajira, in the proximity of Sierra Nevada de Santa Marta.

## Discussion

### Richness and taxonomic diversity of Colombian edible plants

This study provides a comprehensive overview of the edible plant diversity present in Colombia. Results show that the diversity of edible plants in Colombia is remarkable. Half of the species with reported human uses present in the country are edible, for a total of 3805 food plants. This figure far exceeds the previously known numbers for Colombia. Furthermore, this number acquires crucial significance if compared to the data showcased by the WCUP^2^: in fact, the proportion between useful species and edible species at the global level (respectively, 40 292 and 7039 in number) is remarkably lower than the one specific to Colombia. While, according to the Economic Botany Data Collection Standard^39^, at the global level only the 17.4% of useful plants have been recorded as “Human food”^2^, in Colombia this percentage raises up to 53.6%, making this country a global reservoir for edible plant diversity. Nevertheless, only 2457 species are known to be edible in the country, meaning that more than one third of the total diversity of edible plants present in Colombia are unknown or neglected from a gastronomic perspective. This data stresses the untapped potential of Colombian edible plant diversity, and creates an interesting space for future research on the possible reasons behind such gap.

The factors responsible for this notable diversity are many. Among these, it is worth mentioning the unrivalled ecosystem diversity of Colombia: according to the Institute of hydrology, meteorology, and environmental studies (IDEAM, 2017) the country hosts 93 general ecosystems, including 15 coastal ecosystems, as well as 42 terrestrial ones. Some of them are regarded as of global conservation importance, such as the páramos, the Andean forests and the tropical rainforest of the Chocó department^49^, due to the great diversity of life forms they host, as well as their complexity and sensitivity to current environmental changes. What is more, such ecosystem diversity is accompanied by an equally outstanding cultural diversity. According to the National Administrative Department of Statistics^57^ Colombia hosts 87 indigenous peoples and 64 indigenous languages. On the other hand, the Indigenous Organization of Colombia (ONIC) argues that the number of indigenous peoples in the country is, to date, 106^58^. Over the past few decades, there has been increasing recognition of the inextricable link between cultural and biological diversity, and of the countless modalities in which they mutually affect each other^59^. Considering this, Colombia provides a concrete example of how these two dimensions go hand in hand.

Results of the present taxonomic analysis are partially in line with what Diago and García^29^ found: according to these authors, the richest families of edible wild fruit species in Colombia were Fabaceae, Arecaceae, Passifloraceae, Sapotaceae, Moraceae and Melastomataceae. If we consider the totality of Colombian edible plants, results concerning the most important families (i.e., Fabaceae, Asteraceae, Poaceae, Arecaceae, Rubiaceae) differ quite significantly. However, if we only take native edible species into account, results align, highlighting Fabaceae, Moraceae and Melastomataceae as some of the most significantly rich families in edible species. This inconsistency highlights the fact that in Colombia most of the introduced species come from a small number of families, including Fabaceae, Poaceae, Arecaceae. Not surprisingly, these families are among the most important ones for the number of cultivated species, both at the national and at the global level^3^. As the predominance of some introduced cultivated species suggests, the Colombian population has progressively moved away from native foods over the last decades^29^. Numerous native species became NUS due to the progressive transformation of Colombian peoples and their traditional cultural heritage following the advent of European conquerors^60^ and the country's gradual introduction into the globalized trade market of natural products^27^. Today, Colombian gastronomy reflects this country's complex history, with European and African influence from the times of the colonisation, mixed with the rich indigenous cultural heritage and local biodiversity^61^.

According to^62^, despite the unrivalled richness of edible plant species characterising the Colombian territory, today 90% of the natural ingredients marketed in the country are imported. In response to these issues, the Colombian government has recently been investing resources in developing a bioeconomy strategy with the aim of facilitating the future green growth of the country. The Decree^63^, published by the Departamento Administrativo de la Función Pública (Governmental department of Civil Service), provides the first detailed regulatory and legal framework for the sustainable use of non-timber wild edible species. This gives local people the opportunity to commercialise NUS for the first time within a regulated system. Against this background, and based on the abovementioned figures, more in-depth ethnobotanical investigation is needed to identify priority species for revitalization and conservation-through-use initiatives. Numerous examples exist in the literature of how investigations aimed at understanding the relationships between human populations and the natural resources they have traditionally coexisted with and depended on for their subsistence and cultural expression can play an important role in the process of defining conservation priorities^64,65^. Conservation cannot ignore the relationship between the human and non-human dimensions of nature: the daily lives of people following traditional lifestyles are closely connected to the local natural environment (Pei et al. 2020^66^), and the role and perception of a given plant resource within a given socio-cultural context can be of great importance for engaging people in conservation activities and enhancing conservation success^9^.

### Examples of economically important genera and promising species

**Figure 5:**
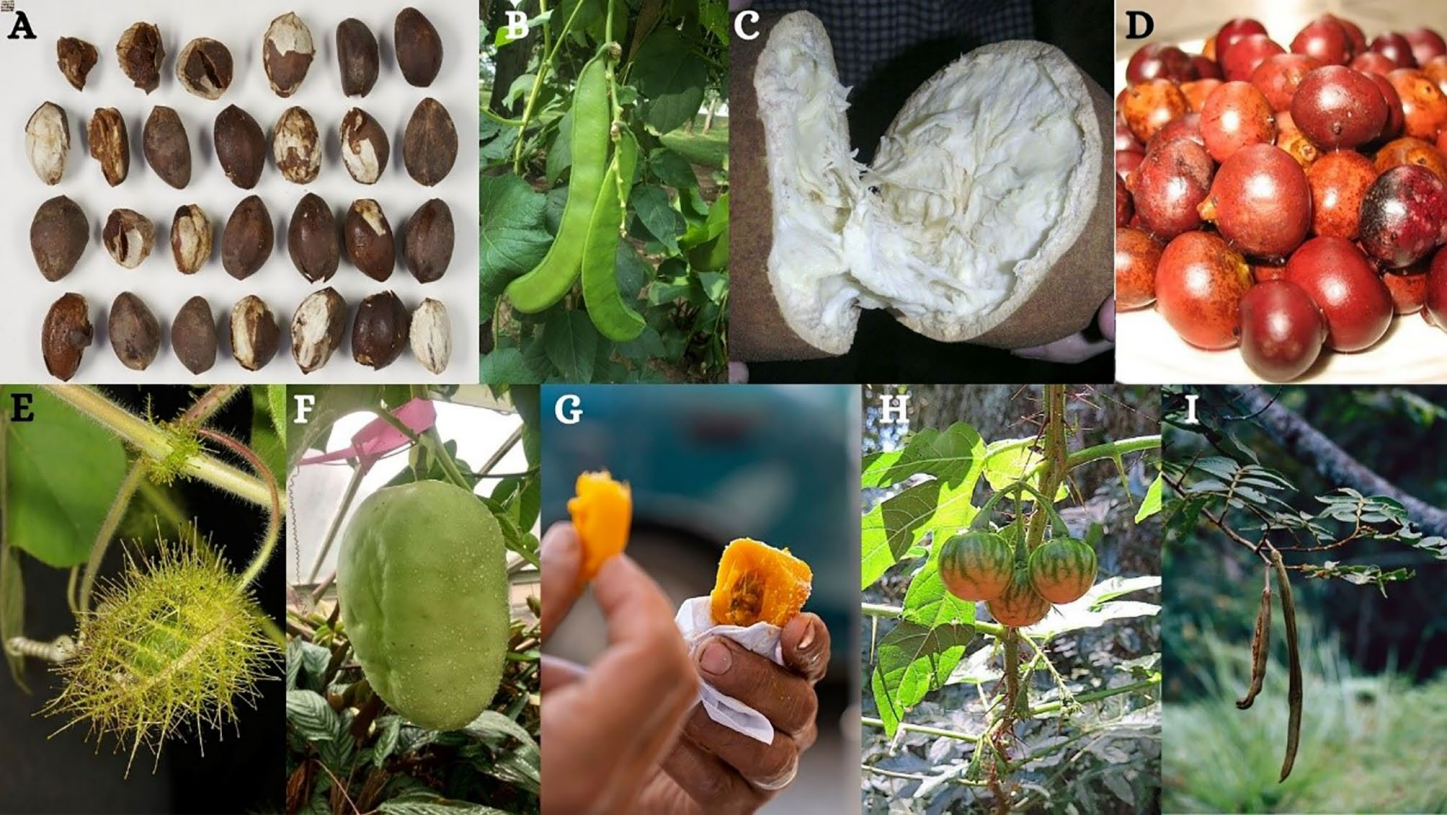
(**A**) *Caryodendron orinocense* (Photograph by Laura Green, distributed under a CC BY open access license via Colplanta, 2021); (**B**) *Canivalia ensiformis* (Photograph by Pradeep Rajatewa, distributed under a CC BY open access license via Colplanta, 2021); (**C**) *Theobroma grandiflorum* (Photograph by BjoernS, distributed under a CC-BY 2.0 license via Wikimedia commons); (**D**) *Bactris guineensis* (Photograoh by Jdvillalobos, distributed under a CC-BY 2.0 license via Wikimedia commons); (**E**) *Passiflora foetida* (Photograph by Ori Fragman-Sapir, distributed under a CC BY open access license via Colplanta, 2021); (**F**) *Passiflora quadrangularis* (Photograph by Rebecca Hilgenhof, distributed under a CC BY open access license via Colplanta, 2021); (**G**) *Bactris gasipaes* (Photograph by David Yela, distributed under a CC-BY 2.0 license via Wikimedia commons); (**H**) *Solanum capsicoides* (Photograph by Dick Culbert, distributed under a CC-BY 2.0 license via Wikimedia commons); (**I**) *Inga edulis* (Photograph by Mauricio Diazgranados, distributed under a CC BY open access license via Colplanta, 2021).

Previous studies have identified several promising species within the Colombian edible plant diversity (e.g.,*Caryodendron orinocense* Karsten, *Erythrina edulis* Triana ex Micheli, *Canavalia ensiformis* (L.) DC. (Fig. 5), *Lupinus mutabilis* Sweet, *Amaranthus caudatus* L., *Theobroma grandiflorum* (Willd. Ex Spreng.) K. Schum. (Fig. 5)), based on their nutritional properties and great resilience and adaptability to a wide range of environmental conditions^67–72^. Here, we want to explore and highlight the four genera that in the present analysis were characterised by having the highest edible species richness and diversity, and conceivably holding great culinary potential and versatility:

An example is the genus ***Passiflora*** (passion flowers or passion vines): although only seven of them are currently cultivated (e.g., *P. antioquiensis* H.Karst. (Curuba antioqueña), *P. caerulea* L. (Pasionaria), *P. edulis* Sims (Maracuyá), *P. ligularis* Juss. (Granadilla), *P. mixta* L.f. (Curubo de indio), *P. quadrangularis* L., *P. tarminiana* Coppens & V.E.Barney (Badea), *P. tripartita* (Juss.) Poir) (Curuba común), other species, including *P. vitifolia* Kunth—known as Granadilla de monte, Granadilla silvestre, Chulupa de mico or Gulupa^73^—are wild harvested for own consumption or sale in local markets. There is substantial morphological variation within the genus (e.g., fruits and flowers’ colour and shape). The fruits can be eaten raw or cooked, or even used to make drinks, as in *P. antioquiensis* (Fig. 5)^73^*.* The pulp is very aromatic, and flowers and leaves are also edible sometimes. In the case of *P. foetida* L. (Cincollaga, Cocorilla) (Fig. 5), leaves are cooked and used as an ingredient in soups. The pulp of fruits is very variable in terms of taste: it can be sweet (e.g., *P. ligularis*), juicy and acid-flavoured (e.g., *P. coccinea* Aubl.) or aromatic and mildly biting (e.g., *P. cumbalensis* (H.Karst.) Harms).

The genus ***Solanum*** can also be considered particularly important from an economic perspective. In addition to *Solanum lycopersicum* L. (Tomato), and *Solanum tuberosum* L. (Potato), this genus has 61 edible species, many of which today have less economic importance, and are known, cultivated and consumed exclusively locally. In Colombia, these are especially present in the Andean region and are still largely unexplored from a taxonomic, agronomic and bromatological perspective. Examples include *S. cajanumense* Kunth, a fast-growing evergreen shrub whose golden-yellow fruits are eaten fresh when fully ripe; *S. capsicoides* All. (Fig. 5), whose poisonous fruits can be eaten when roasted or cooked; and *S. pectinatum* Dunal—commonly known as Huevo de gato, Naranjuelo or Toronja^73^—whose pale orange flash, characterized by a sweet-acidic flavour, is delicious when cooked with sugar (Food Plants International, 2021^74^).

***Inga*** constitutes another excellent example of promising genera: its edible fruits are very popular throughout South America, where they are usually wild harvested^75^. Out of 84 species found in Colombia, only six are currently cultivated (i.e., *I. densiflora* Benth. (Guamo macheto), *I. edulis* (Guamo), *I. feuillei* DC. (Guabo), *I. ornate* Kunth (Guamo), *I. spectabilis* (Vahl) Willd. (Guamo macheto) and *I. vera* Willd. (Guamo)). *Inga edulis* Mart. (Fig. 5)—also known as Churimo or Guabo—is the best known and most consumed species. Both the seeds and the white, jelly pulp surrounding them can be eaten. The pulp is characterised by a sweet and highly aromatic taste^76^ and it is usually eaten raw. The seeds are eaten cooked, usually boiled or roasted, as in the case of *I. ilta* T.D.Penn (Guamo de semilla). When immature, they can also be eaten raw, blanched and salted, and added to salads (Food Plants International, 2021^74^). Inga trees also hold great environmental value: they are commonly placed in coffee or cacao plantations to provide shade to the surrounding environment^77,78^.

Finally, ***Bactris*** counts 28 edible species in Colombia, and all of them are native. However, only *B. gasipaes* Kunth (Chontaduro, Cachipay, Pipire) (Fig. 5) is currently cultivated. Fruit of most species are inedible raw^79^. They are usually boiled in salted water for thirty to sixty minutes and eaten as a vegetable (Food Plants International, 2021^74^). The pulp is characterised by a nutty flavour and a floury texture, as well as remarkably high nutritional properties due to their great protein and carbohydrate content. Fruits can also be made into a flour and baked into bread, cakes, and other processed foods^79^, Food Plants International, 2021^74^). Seeds can be consumed raw^80^, as nuts, as in the case of *B. major* Jacq. (Lata arroyera, Albarica, Uvita de tigre) and *B. gasipes*. The palm heart of some species (e.g., *B. riparia* Mart., *B. corossilla* H.Karst.) is also eaten raw, in salads, or cooked. Finally, the fruits of *B. guineensis* (L.) H.E.Moore (Lata de corozo) (Fig. 5) can be fermented and used to produce a drink, which in Colombia is known as “Chicha de corozo”^81,82^.

### Distribution of occurrence records

Results clearly show how occurrences records are unequally distributed among Colombian edible species. This constitutes a substantial limitation to the study of the distribution patterns of the Colombian edible flora and can be mainly attributed to the lack of scientific coverage of some areas of the country^49^. In fact, inequalities in the distribution of georeferenced records does not only apply to species diversity but also to entire Colombian departments and bioregions. Linear regressions revealed a significant correlation (p < 0.001) between species richness and number of georeferenced records within Colombian departments, making it possible for us to argue that while some of the striking differences in species richness between Colombian departments (Fig. 2D) can be attributed to environmental and anthropogenic factors, other may be the result of the lack of adequate on-site investigation.

These results are consistent with what^49^ reported on the distribution of useful plants across Colombian bioregions: while exhaustive sampling in areas such as the Andean region allows the comprehensive understanding of the edible flora of the Andean humid forest, the Andean dry forest and the Páramo bioregions, the regions of Caribe and Llanos (Cf. Fig. 1), to date, remain largely unexplored. This represents a substantial gap to the reliability of the present figures.

As^40^ point out, both historical and security factors may have contributed to an unbalanced sampling effort across these regions. The internal conflict that Colombia has witnessed over the past six decades has prevented scientists from carrying out field work in several parts of the country, including the departments of Cesar, Norte de Santander and Arauca^40^. Both the Llanos and Caribe areas are known for violent episodes and illegal activities, explaining the scarcity of scientific studies there, and the scarcity of recorded edible plant species. Therefore, entire bioregions, located in such under-surveyed areas of the country, are likely to be underrated by the present results. These include the Caribbean dry forests and páramo, the humid and dry forests of the Llanos, the Amazonian humid forest and the savannas of the Orinoquia region.

### Biogeographic patterns of Colombian edible plants

A significant level of geographic specificity was found in the distribution of Colombian edible species across 33 departments, with most of the species only recorded within five of them or fewer, stressing the remarkable biological difference between the numerous bioregions and ecosystems across the country. This figure is confirmed by the CWE analysis (Fig. 4C), which instead of revealing few clear hotspots for narrow-distribution edible plants, such as in the case of SR and WE (Fig. 4A, B), displays numerous cells (10 × 10 km^2^) of high endemic value in several areas of the country. In fact, areas that were not highlighted by the SR metric, such as the regions of Amazonia, Llanos and Caribe, are shown to be of great conservation importance due to the presence of unique species. This suggests that, although bioregions such as the Andean humid and dry forests have recorded the highest amount and diversity of edible species, especially within departments of Antioquia and Boyacá, such species were not characterised by restricted distribution ranges. In fact, these regions contain the main agricultural areas of Colombia and are therefore characterised by the presence of cultivated species with broad distributions. Considering this, the CWE metric is key to emphasise the hidden biological importance of unexplored Colombian bioregions, as well as to recognise the limitations of our current understanding of the biogeographic distribution of local edible plants.

#### Species richness

The Colombian Andes form part of the Tropical Andes, which extend across the north of Chile, Argentina, Bolivia, Peru, Ecuador, Colombia and Venezuela for over 1.5 million km^2^, between the latitudinal range of 11° N to 30° S. They are characterised by an elevational range of approximately 500 to 6000 m a.s.l. (Bax and Francesconi 2019^83^). According to Meyers et al. (2000)^102^, the Tropical Andes support approximately 45 000 plant species, with nearly half of them being endemic to the Andean ecoregion. However, due to increasing human-driven alterations of natural ecosystems, as well as to the progressive impact of climatic variations, ecosystems such as the Tropical Andes are nowadays recognised as one of the most critically threatened ecoregions in the tropics^84^. This constitutes a particularly serious hazard to the native endemic edible species inhabiting the region, characterised by extremely specific habitat needs. In light of this, Colombian Andes are expected to lose a significant proportion of their native plant diversity by reason of environmental degradation^84,85^. This would represent a significant loss not only for the biological heritage characterising the region, but also for its socio-cultural one, embedded in local traditional agricultural practices and gastronomy. Andean bioregions and the edible flora characterising them must therefore be protected and further investigated from an ethnobotanical perspective, in order to understand the mechanisms and socio-cultural practices underlying their remarkable gastronomic heritage.

#### Corrected weighted endemism

A significantly high density of narrow-distribution edible plants was recorded in the northern part of the Huila department. Huila may be considered one of the richest regions of Colombia in terms of plant biodiversity, due to its great variety of ecosystems, from páramos on top of the mountains to extensive areas of tropical dry forest and rainforest. About 120 000 hectares of the department are localised within the páramo biome, with elevations ranging between 2900 and 5000 m a.s.l. Notably, the area has been described as one of the richest in the country in terms of diversity of the genus *Passiflora*^86^. Another interesting hotspot for its unique edible flora can be spotted in the department of La Guajira (Caribe), in the vicinity of the Sierra Nevada de Santa Marta (SNSM), a UNESCO Biosphere Reserve. According to Durán-Izquierdo and Olivero-Verbel^87^, the SNSM can be considered as the most irreplaceable nature reserve in Colombia, due to its extraordinary ecological diversity. However, today SNSM’s precious biomes, as well as the ecosystem services they provide, are increasingly being jeopardized by anthropogenic activities such as mining, agricultural expansion and tourism^87^. High concentrations of edible species with restricted distribution can also be found in the humid forests of Amazonia and Llanos, encompassing the departments of Amazonas, Caquetá and Guainía. These are some of the least accessible areas of the country due to the poor road coverage^49^. High species density cells occur especially around four national parks: Parque Nacional Natural Yaigoje Apaporis, Parque Nacional Natural Cahuinarí, Reserva Nacional Natural Nukak and Reserva Nacional Natural Puinawai. These places are focal points of biocultural diversity for the whole country, and over the years they all have witnessed, and to a large extent still witness, effective examples of indigenous resistance against mining expansion^88,89^. According to Bystriakova and colleagues^49^, only 8% of the Amazonian humid forest has been displaced by human activities. However, despite the institution of protected areas, deforestation and land use change still represent major threats to these sites. The insufficient sampling effort in these regions, together with the increasing pressure on natural resources, could result in the loss of a consistent portion of edible plant diversity which to date has still not been fully documented. One final hotspot is visible in the Nariño department, across the humid forests of the Pacific and the Andean regions. In particular, the highest concentration of edible species is scattered along the banks of the Mira River. In addition to such bioregions, the department also includes Andean humid and dry forests, páramos, and mangroves. Such biological diversity corresponds to an equally rich cultural diversity, with 17.8% of its population being Afro-Colombian and 15.7% belonging to various indigenous peoples^57^. Therefore, Nariño can be regarded as a remarkable hotspot for the Colombian biocultural diversity.

### Food security & food sovereignty

The uneven distribution of occurrence records among edible species—with introduced cultivated species registering outstanding numbers of georeferenced records compared to native ones—closely reflects the process of establishment and expansion of large-scale plantations of commercial species, primarily destined for the international market. A concrete example is provided by Hurtado-Bermudez and colleagues^25^, whose work examined the increasing spread of sugarcane plantations (*Saccharum officinarum*) across the regions of Magdalena and Cauca. According to the authors, such expansion has led to increasing land dispossession, farmers displacement and food insecurity in the regions^25^. Indeed, Colombia reports one of the highest rates of inequalities regarding land ownership^90^, which often goes hand in hand with decreasing food security and sovereignty. In 2015, the prevalence of food insecurity in rural households in Colombia was 54.2%^91^. Moreover, targeted quantitative examination of indigenous and Afro-Colombian households revealed a much higher prevalence of food insecurity compared to national figures, ranging between 70 and 85%^25^. Studies demonstrate how large-scale industrial agricultural systems have negatively impacted Afro-descendant and indigenous peoples from a cultural and economic perspective, causing significant socio-environmental transformations, as well as progressive loss of autonomy over their territory and consequent impoverishment^92–94^. In response, we stress the potential of Colombian edible NUS for tackling these issues. We advocate the need to build locally controlled food systems, rooted in the local environment, culture and traditions, and the urgent need to create new sustainable livelihoods for local peoples, based on the cultivation and commercialisation of native edible plants. NUS are highly promising resources for agriculture, novel products and nutritional improvement^15^. It is therefore vital to promote new agricultural models that revalorise their use and provide an alternative approach to the spread of monocultures and intensive farming.

### An example of best practice: the Guáimaro (*Brosimum alicastrum* Sw.)

**Figure 6:**
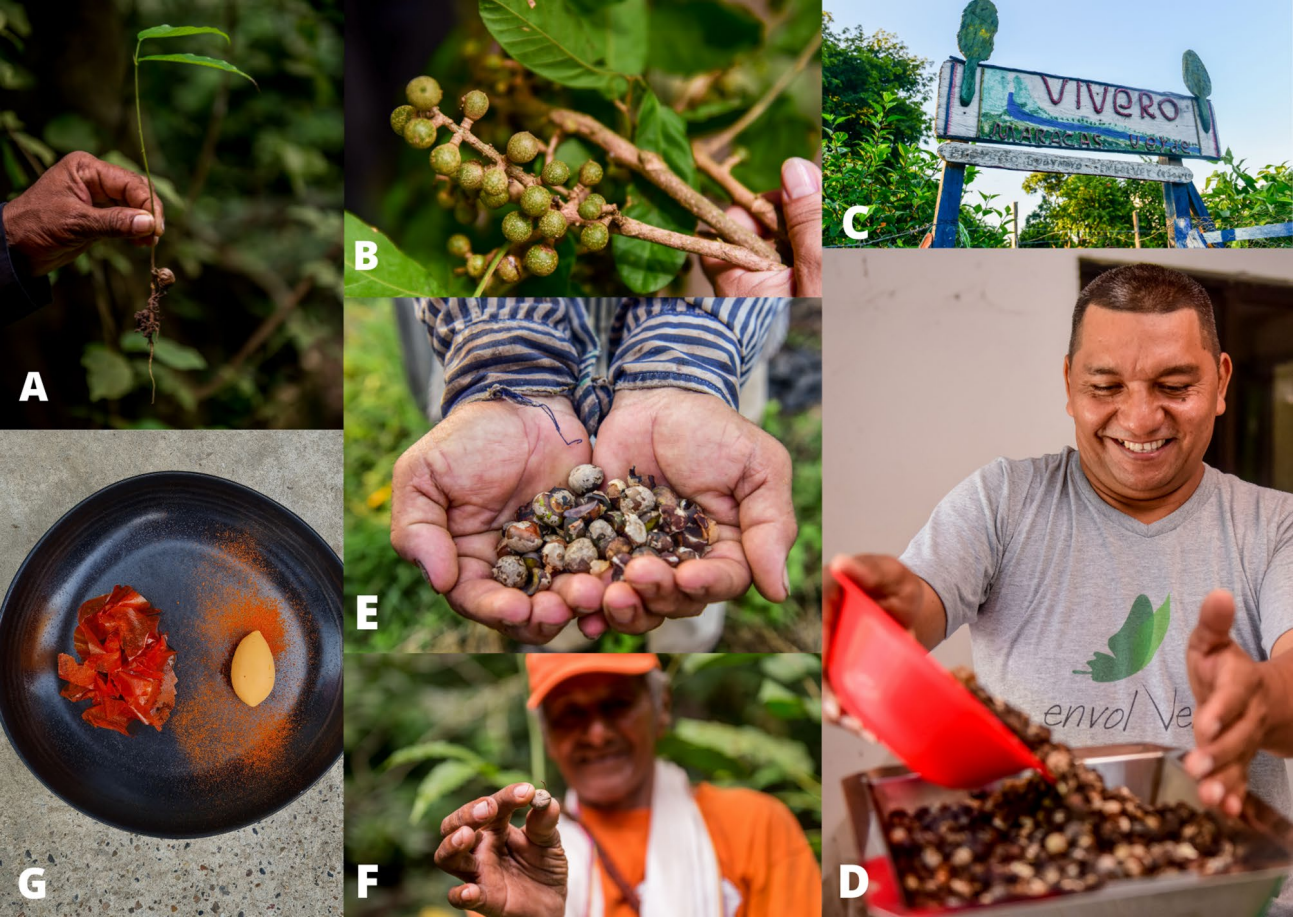
(**A**) *Brosimum alicastrum* germination from seed; (**B**) *Brosimum alicastrum*’s fruit; (**C**) Community’s plant nursery; (**D**) Community member of Becerril processing Guáimaro seeds in the local facility; (**E**) Guáimaro seeds; (**F**) Guáimaro seed showed by a community member in Becerril; (**G**) Dessert prepared using Guáimaro (*B. alicastrum)* by Chef Jaime Rodríguez Camacho at Celele restaurant in Cartagena. Credits: A-F) N. Plata; G) J. Rodriguez Camacho.

The Guáimaro (*Brosimum alicastrum* Sw.), also known as Caucho, Lechero and Sande ^73^ is a wild edible NUS belonging to the Moraceae family, native to tropical dry forests in Mesoamerica and the Caribbean^95,96^ (Fig. 6). Due to its great environmental, social and economic potential, the Guáimaro was identified as a priority species by the Useful Plants and Fungi of Colombia (UPFC) project in one of its three pilot areas in the country: Becerríl (Cesar). Besides being an important ecological indicator of the health of the forest, this species is of great importance for the protection of soil and water bodies, as well as for the feeding of wildlife^95^. What is more, the Guáimaro is characterised by great nutritional qualities, such as a high carbohydrates’ content and antioxidant activity^97^, as well as a great culinary versatility. The seed (Fig. 6E, F) used to be a staple food for prehispanic cultures such as the Yukpa indigenous peoples^98^. It can be eaten raw, boiled, roasted or made into flour, which can be used as an ingredient to improve the nutritional properties of traditional dishes^97^. Nowadays, it is estimated that only 5% of the original Guáimaro forest cover remains^99^. In the area of Becerril, main causes of disappearing of this species include coal mining, sowing of African palm for oil extraction and bovine and ovine extensive farming^99^. Promoting its revitalisation through sustainable consumption and commercialisation practices in the community of Becerril has proven to be an effective tool for ecological conservation and forest restoration: a community-based facility for local processing of the Guáimaro fruits was established (Fig. 6D), and direct sale of the processed nuts increased in local markets. Additionally, commercial connections were built between farmers in Becerril and restaurants across the country through UPFC and local partnering NGOs (Fig. 6G), giving rise to a new demand for this NUS and increasing its perceived value. Finally, in addition to economic and environmental benefits, increase in the consumption of Guáimaro-based flour in traditional preparations such as *arepas*, *empanadas*, soups and *enyucados*, has the potential to strengthen the food security of the community.

## Conclusion

By disclosing the richness, diversity, and potential of Colombian edible plant diversity, and identifying current knowledge gaps at the geographic level, the present analysis constitutes a strong empirical basis for directing further research efforts targeting least explored areas of Colombia. The in-depth characterisation of Colombian edible plant resources is important to achieve their effective protection, to guarantee their survival and to encourage their recovery and valorisation. This process requires the joint forces of numerous disciplines, ranging from taxonomy, biogeography, ethnobotany and bromatology, together with the generation of more complete and detailed information on the population size, distribution range and threats monitoring of species^100^. This study has contributed to the preliminary characterization of edible plant resources in Colombia both from a taxonomic and biogeographic perspective. At the taxonomic level, Colombian edible plants cover an unrivalled variety of families, genera and species, many highly localised. Thanks to its unique and diverse natural ecosystems, as well as to their exhaustive sampling, the Andean region scored the highest number of edible species. On the other hand, regions like the Amazon, the Caribbean, and Llanos still remain poorly explored from a scientific standpoint and should therefore be prioritised for future, focussed research.

Today, the preservation of Colombian NUS and the encouragement of their use are more crucial than ever. After more than six decades of internal conflict, the country is currently going through fast changes, which will determine the fate of its natural resources. Agricultural expansion and urban development are leading to deforestation and habitat loss, resulting in unprecedented levels of biodiversity erosion. Local NUS hold great potential for supporting local livelihoods and developing a bioeconomy based on the sustainable use of local natural resources. Therefore, further targeted ethnobotanical, bromatological and agricultural studies are urgently needed to achieve the full characterisation of these resources, and direct future prioritisation efforts toward their revitalisation and conservation-though-use.
